# Phylogenetic placement of the unusual jumping spider *Depreissia* Lessert, and a new synapomorphy uniting Hisponinae and Salticinae (Araneae, Salticidae)

**DOI:** 10.3897/zookeys.549.6171

**Published:** 2016-01-05

**Authors:** Wayne P. Maddison, David R. Maddison, Junxia Zhang, Tamás Szűts

**Affiliations:** 1 Beaty Biodiversity Museum and Departments of Botany and Zoology, University of British Columbia, Vancouver, British Columbia, V6T 1Z4 Canada; 2 Department of Integrative Biology, Oregon State University, Corvallis, OR 97331, USA; 3 Department of Entomology, University of California, Riverside, Riverside, CA 92521, USA; 4 Department of Zoology, Savaria University Centre, University of West Hungary, Szombathely, H-9700 Hungary; 5 Department of Entomology, California Academy of Sciences, San Francisco, CA 94118, USA

**Keywords:** Jumping spiders, Salticidae, phylogeny, systematics, Cocalodini

## Abstract

The relationships of the unusual salticid spider *Depreissia* from central Africa and Borneo have been difficult to resolve, obscured by its highly modified ant-like body. Phylogenetic analysis of the gene 28S strongly supports its placement outside the major clade Salticinae and within the clade of cocalodines, spartaeines and lapsiines, with weaker support for a relationship with the cocalodines in particular. Excluding the genus from the Salticinae is supported also by the presence of a median apophysis on the male palp, and by the lack of a cymbial apical groove cradling the tip of embolus, which is newly presented here as a synapomorphy of Hisponinae plus Salticinae.

## Introduction

The strange salticid spider *Depreissia* Lessert, 1942 is known from only a few specimens representing two species, *Depreissia myrmex* Lessert, 1942 from Africa (Lessert 1942, Wesołowska 1997, Szűts and Wesołowska 2003) and *Depreissia decipiens* Deeleman-Reinhold & Floren, 2003 from Borneo (Deeleman-Reinhold and Floren 2003). These species have been noted for their unusual structures, with an extraordinarily long pedicel and strange constrictions and dimples on the carapace (Lessert 1942, Wesołowska 1997, Szűts and Wesołowska 2003, Deeleman-Reinhold and Floren 2003, Deeleman-Reinhold et al. 2016), which given them the appearance of ants or wasps (Deeleman-Reinhold et al. in press). These autapomorphic features have not helped placing *Depreissia* phylogenetically, however. Both Lessert (1942) and Wesołowska (1997) suggested a relationship with *Leptorchestes* Thorell, 1870, although both expressed difficulty in placing *Depreissia* in any known group, and noted the unusual eye arrangement typical for lyssomanines and *Athamas* O. P.-Cambridge, 1877.

The major clade Salticinae (*sensu* Maddison in press), including over 90% of the species of jumping spiders, is delimited by several morphological synapomorphies, including the lack of a tarsal claw in the female palp, and is well supported by molecular data (Maddison and Hedin 2003, Maddison et al. 2014). Outside of it are the lineages known as “basal salticids”, including hisponines, spartaeines, lapsiines, cocalodines, eupoines and lyssomanines (Wanless 1980, 1984, Maddison and Needham 2006, Maddison et al. 2007, Maddison 2009). Insofar as there are relatively few basal lineages, the recognition of a new one could be of special interest for interpreting the early evolution of salticids (see Maddison and Needham 2006: 38).

In this paper we present both molecular and morphological evidence that supports the placement of *Depreissia* outside the Salticinae, possibly as a close relative of the cocalodines. Deeleman-Reinhold et al. (2016) has independently discovered morphological evidence supporting the same placement.

## Methods

### Taxon sampling for molecular phylogeny

**Figure 1.**
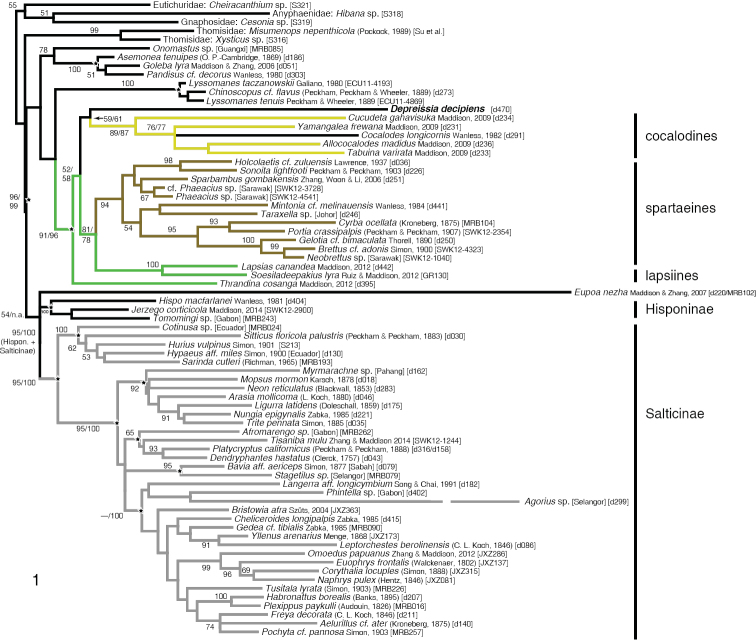
Maximum likelihood phylogenetic tree from 28S sequences, constrained as described in text. Branches labelled by bootstrap percentages from 500 replicates, with selected branches also showing (after “/”) bootstrap percentages for analysis with *Eupoa* and *Agorius* removed. Branch length of *Agorius* abridged to 50% of its actual length. Voucher specimen codes appended in brackets. Cocalodine, spartaeine and lapsiine lineages are colored as in Maddison et al. (2014). Stars mark clades constrained except for the freedom of *Depreissia* and *Agorius*.

A juvenile of *Depreissia decipiens* (with labels “Kinabalu NP, My 6°5'N 116°33'E Poring Hot Springs, A. lagenocarpa 13 A. Floren. 16.9.2006. B 14”, and “WPM DNA voucher d470”) was supplied by Christa Deeleman-Reinhold. Data for 28S from this specimen was added to data from 72 taxa included in the analysis of Maddison et al. (2014), selected to cover the breadth of salticid diversity, and because their 28S sequences were fairly long. Information on those specimens and sequences can be found in Maddison et al. (2014) by following the voucher codes in brackets in the taxon names of Fig. 1.

### Sequencing

DNA was extracted from the entire body of a single immature male using a Qiagen DNeasy Blood and Tissue Kit. The specimen was not ground, but several holes were torn into the body wall to allow better penetration of buffers and enzymes. The fragment of 28S was amplified using the Polymerase Chain Reaction on an Eppendorf Mastercycler Thermal Cycler ProS, using TaKaRa Ex Taq and the basic protocols recommended by the manufacturer. Two PCR reactions were conducted, one with primers LS58F and LS998R, the other with primers NLF184/21 and LS1041R (Maddison 2012), both using cycling reaction CI in Maddison (2012: 573). The amplified products were then cleaned, quantified, and sequenced at the University of Arizona’s Genomic and Technology Core Facility using a 3730 XL Applied Biosystems automatic sequencer. Initial base calls and assembly of the four chromatograms into one fragment were made with Phred (Green and Ewing 2002) and Phrap (Green 1999) as orchestrated by Mesquite’s Chromaseq package (Maddison and Maddison 2014, 2015) with subsequent modifications by Chromaseq and manual inspection.

### Sequence alignment and phylogenetic analysis

Automatic multiple sequence alignment was performed by MAFFT (Katoh et al. 2002, 2005), run via the align package of Mesquite (Maddison and Maddison 2015). Alignment used the L-INS-i option (--localpair --maxiterate 1000).

Phylogenetic analyses using maximum likelihood were run using RAxML version 7.2.8alpha (Stamatakis 2006a, b). RAxML runs assuming the GTRGAMMAI model were performed with 100 search replicates, to seek maximum likelihood trees. In addition, likelihood bootstrap analysis was performed with 500 bootstrap replicates, each involving a single search replicate. We performed both constrained and unconstrained analyses. The unconstrained analyses used only the data at hand: the 28S gene in the 73 taxa. This unconstrained analysis has the flaw that it fails to consider data from other genes and other taxa, which have convincingly demonstrated the monophyly of many major clades of salticids. Because the gene 28S does not properly resolve some of the deeper relationships of the family (Maddison et al. 2014), this analysis places *Depreissia* on a faulty background. We therefore also performed analyses that constrained the tree to enforce monophyly of some groups, thus placing *Depreissia* on a well-supported salticid phylogeny. The full constraints analysis holds the following groups monophyletic as they are well supported by multigene analyses (Maddison et al. 2014): Salticidae, *Lyssomanes* + *Chinoscopus*, *Asemonea* + *Goleba* + *Pandisus*, spartaeines + cocalodines + lapsiines, Hisponinae, Salticinae, Amycoida, Salticoida, *Bavia* + *Stagetilus*, Astioida, Marpissoida, and Saltafresia. Relationships within those groups were free to vary, and the placements of *Depreissia* and *Agorius* Thorell, 1877 were free to move throughout the tree, including within the otherwise constrained clades. Thus, even though these clades were constrained, they could have had bootstrap percentages less than 100% if *Depreissia* or *Agorius* had wandered inside or outside them. Another less constrained analysis enforced the monophyly of only the Salticidae. Because *Eupoa* Żabka, 1985 and *Agorius* have unusual 28S genes and are unstable in analyses (Maddison et al. 2014), they were excluded in some variant analyses.

### Morphology

Structures of *Depreissia myrmex* were studied from a specimen deposited in the HNHM (Hungarian Natural History Museum, curator Dr. László Dányi, label data: Araneae-4515: *Depreissia myrmex* Lessert, 1942: Republic of Congo, Kindamba, Meya, Bangu forest, 1963., leg: J. Balogh & A. Zicsi, det.: Wanda Wesolowska („HNHM Nr. 325”) 3 50'S, 14 30'E). The specimen was examined with an Olympus SZ60 microscope; images were taken with Leica DM2700 M, Nikon Eclipse and Zeiss JENAVAL microscopes. For examination of internal structures, the palp was immersed in absolute ethanol then temporarily cleared with a solution of methyl salicylate (synthetic version of wintergreen oil), fixed with a modified version of Coddington’s temporary mount (Coddington 1983), and photographed with an attached Nikon D300S camera using Helicon Remote®. Multiple stacked images were montaged using the program Helicon Focus®.

Specimens illustrated in the figures are listed in the Appendix. Abbreviations for collections are UBC-SEM (University of British Columbia, Spencer Entomological Collection), HNHM (Hungarian Natural History Museum), CAS (California Academy of Sciences) and ZMUC (Zoological Museum, University of Copenhagen).

### Data resources

The data underpinning the analyses reported in this paper, and the resulting trees, are deposited in the Dryad Data Repository at http://dx.doi.org/10.5061/dryad.gd501.

## Molecular phylogenetic results

The new sequence for 28S for *Depreissia decipiens* has been deposited in GenBank with accession number KT462690.

The maximum likelihood phylogenetic tree found in the fully constrained analysis is shown in Fig. 1. *Depreissia* is placed as sister to the cocalodines, though the bootstrap support appears low. Its placement within the larger clade of cocalodines + spartaeines + lapsiines is, however, strongly supported (bootstrap percentage 91, 96 if the unstable *Eupoa* and *Agorius* are excluded from the analysis). The bootstrap support for the Salticinae is high, 95% (100% if *Eupoa* and *Agorius* are excluded), indicating that *Depreissia* is not a salticine.

The partially constrained analysis (only Salticidae enforced) gave considerably lower bootstrap values, with 62% support for *Depreissia* within the cocalodines, 63% for *Depreissia* within the larger clade of cocalodines + spartaeines + lapsiines, and 52% support for the Salticinae. However, a more detailed inspection of the trees from bootstrap replicates shows that support for placement of *Depreissia* remains strong. Of the 500 bootstrap replicates, 445 (89%) place *Depreissia* either with the cocalodines or with the broader group of cocalodines + spartaeines + lapsiines, in some with *Eupoa* included also. Of the remaining replicates, *Depreissia* is placed outside of the Salticinae (sensu Maddison in press) in 47. This leaves 11 replicates, of which 5 place *Depreissia* among other basal salticids (e.g., the lyssomanines). Five associate *Depreissia* with the likewise ant-like *Agorius*, but that is a result of *Agorius* moving outside the Salticinae, not *Depreissia* moving inside. Only a single replicate places *Depreissia* inside the Salticinae, as sister to the amycoids. Thus, the bootstrap support for *Depreissia* being outside the Salticinae is 99.8%.

The unconstrained analysis also provides strong support for *Depreissia*’s placement, with 100% support for the exclusion of *Depreissia* from the Salticinae and 86% support for a placement near the cocalodines. Of the 500 bootstrap replicates, 429 place *Depreissia* either with the cocalodines or with the broader group of cocalodines + spartaeines + lapsiines, in some with *Eupoa* or *Hibana* Brescovit, 1991 included also. Of the remaining 71 replicates, 69 clearly place *Depreissia* among other non-salticine groups (sometimes the lyssomanines). The last 2 trees associate *Depreissia* with *Agorius*, with both being outside the Salticinae.

### Morphological evidence

**Figures 2–7.**
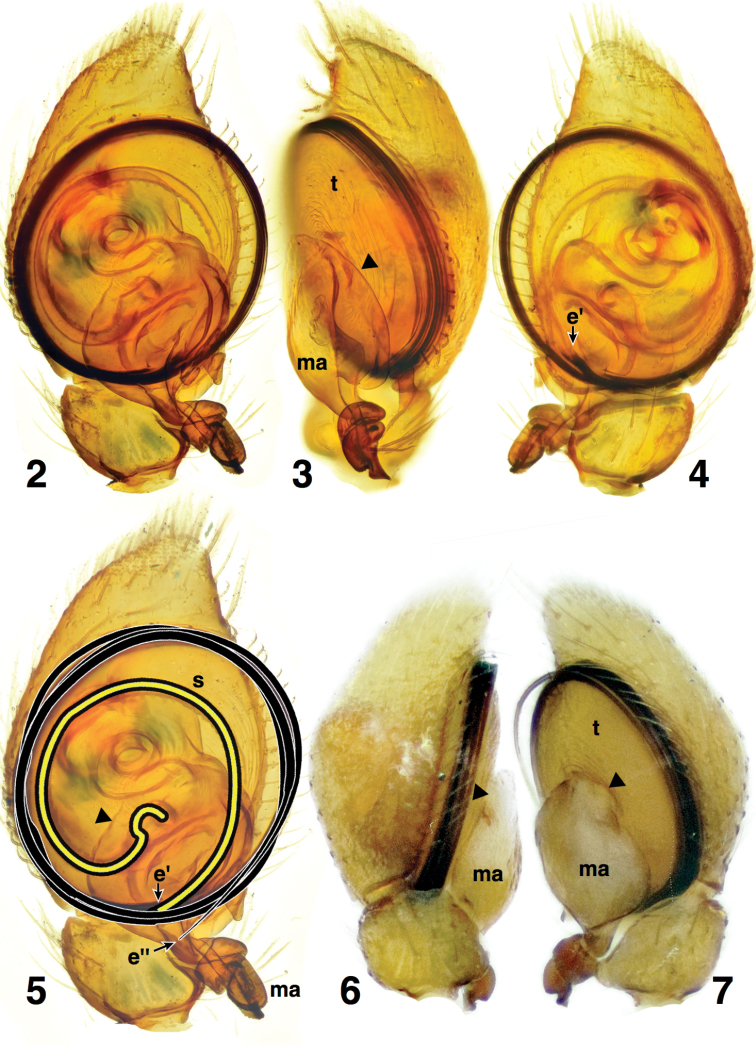
Male palp of *Depreissia myrmex* **2** Cleared palp, ventral view. **3** Retrolateral view **4** Dorsal view **5** Ventral view, highlighting the path of spermaphore (yellow) and embolus (black) **6** Left palp, prolateral view **7** Right palp, prolateral view. Abbreviations: **e’** = origin of embolus; **e’’** = end of embolus; **ma** = median apophysis; **s** = spermophore; **t** = tegulum. Triangle marks base of median apophysis.

The embolus of *Depreissia* wraps around the round bulb, appearing merely as a peripheral black edge (Wesołowska 1997, Deeleman-Reinhold and Floren 2003, Szűts and Wesołowska 2003). In *Depreissia myrmex* (Figs 2–6, 9–10), the embolus originates at about 6 o’clock (Figs 4, 5e’), then loops three times around the bulb to end near the origin (Fig. 5e’’). The spermophore is fairly narrow throughout (Fig. 5s).

**Figures 8–13.**
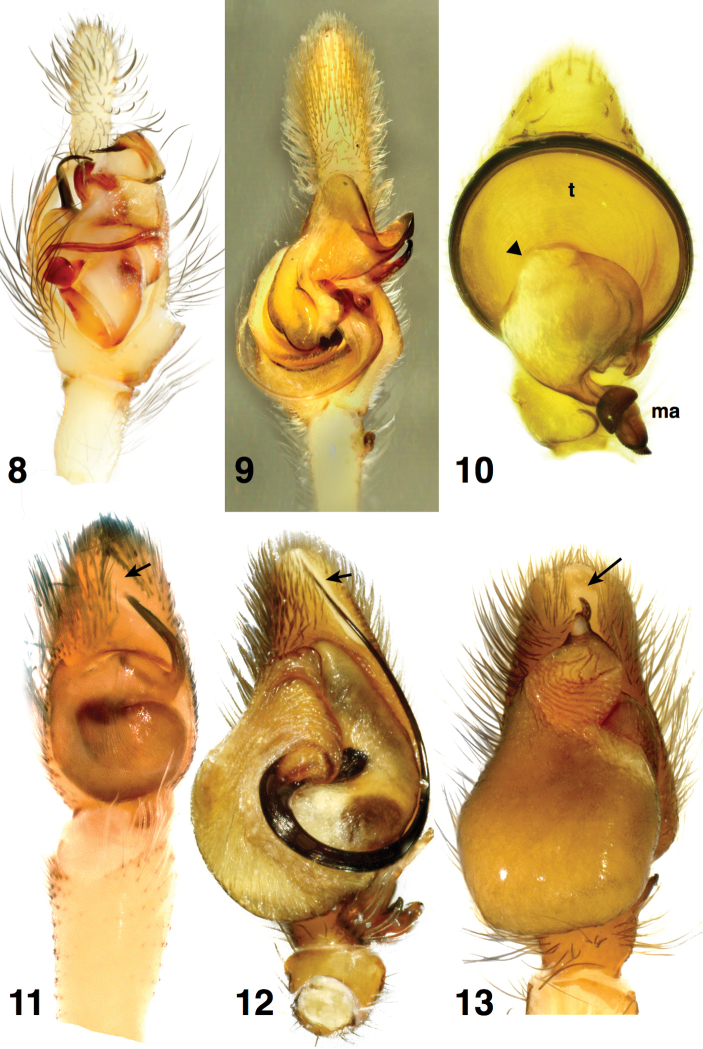
Male palps to show absence (**8–10**) or presence (**11–13**) of the cymbial apical groove (a depression in the cymbium that cradles the tip of the embolus, marked with an arrow) **8** *Lyssomanes taczanowskii* Galiano, 1980 **9** *Cocalodes papuanus* Simon, 1900 **10** *Depreissia myrmex* Lessert, 1942 **11** *Tomocyrba ubicki* Szűts & Scharff, 2009 **12** *Hispo sulcata* Wanless, 1981 **13** *Phidippus audax* (Hentz, 1845). Triangle marks base of median apophysis (**ma**).

Many salticids outside of the Salticinae have a median apophysis on the palp, the loss of which is a synapomorphy of the Salticinae (Maddison and Needham 2006, Maddison et al. 2007, Maddison 2009, Maddison in press). The median apophysis arises from the tegulum, and is usually separated from it by a membrane (e.g., Maddison and Needham 2006, Maddison et al. 2007, Maddison 2009). On the tegulum of *Depreissia* is a large complex apophysis (Wesołowska 1997: fig. 3, Deeleman-Reinhold and Floren 2003: fig. 3), referred to as a tegular projection by Deeleman-Reinhold and Floren (2003). It arises from the middle of the face of the tegulum, expands to a bulbous projection, then narrows and twists at its heavily sclerotized tip (Figs 2–7, 10, ma). This projection is quite distinct from the tegulum, separated from it by a narrow waist (Figs 3, 6, 10, triangle), and is apparently surrounded by a membranous area (Figs 6, 7, 10, triangle). No tegular apophysis with these qualities is seen in salticids except the median apophysis of basal salticids. We therefore judge it to be a median apophysis, which places *Depreissia* outside the Salticinae.

Deeleman-Reinhold et al. (2016) also conclude that a median apophysis is present, but they interpret it to be a smaller sclerite. While we interpret the entire “tegular projection” to be the median apophysis, Deeleman-Reinhold et al. (2016) interpret the projection to be a lobe of the tegulum, and only the dark tip of the projection to be the median apophysis (“tip-only”). Our interpretation is based on the fact that the tegular projection is separated by a narrow and membranous neck from the rest of the tegulum, matching the quality of the tegulum-median apophysis boundary as seen in other salticids. We judge the “tip-only” interpretation as less parsimonious, because it implies a new distinct feature not seen in other salticids (an isolated ventral lobe of the tegulum) as well as a loss of the membrane at the base of the median apophysis.

Our interpretation suggests that the median apophysis is unusually large, but cocalodines have remarkably large median apophyses (Maddison 2009), which may indeed be a synapomorphy uniting *Depreissia* with the cocalodines. Except for *Cucudeta gahavisuka* Maddison, 2009, cocalodines have a median apophysis whose length is as great as or greater than half the diameter of the bulb (see figures in Maddison 2009). Lapsiines, spartaeines, and hisponines have smaller median apophyses, with the exception of *Thrandina* Maddison, 2006 (Maddison 2012); the size in lyssomanines is variable (see e.g. Logunov 2014).

Another character that clearly supports the non-salticine status of *Depreissia* is the lack of a depression in the cymbium cradling the tip of the embolus (see Figs 8–13). In hisponines and salticines, the tip of the embolus falls in a consistent place over the tip of the cymbium, resting in a shallow, elongated groove in the tegulum (Figs 11–13, arrow). Among salticids outside the hisponines and salticines, the groove is absent (Figs 8–9). It is also lacking in *Depreissia* (Figs 2–4, 7, 10). Outside the salticids it is lacking in the apparently closely-related crab spiders (Ramírez 2003, 2014), but it is present in anyphaenids (Ramírez 2003) and castianieirines (Ramírez 2014). This groove, which occasionally extends to the dorsal side, was called the cymbial groove by Szűts and Scharff (2005), and is one of several cymbial grooves of Ramírez (2014), the cymbial apical groove. The presence of a cymbial apical groove cradling the embolus is concordant with molecular evidence that supports the clade consisting of hisponines plus salticines (Maddison et al. 2014). Its absence is therefore evidence that *Depreissia* is a non-salticine.

While the morphological data support *Depreissia* as a non-salticine, the morphological data neither rule out its being a cocalodine, nor give good support for its placement with the cocalodines in particular. This is not surprising, as there are no known unambiguous morphological synapomorphies either for the cocalodines (Maddison 2009) or for the larger group including lapsiines and spartaeines.

## Conclusion

Based on both the morphological and molecular evidence, we conclude that *Depreissia* is outside the major clade Salticinae. Furthermore, the molecular evidence suggests that it can be provisionally associated with the cocalodines, and the morphological evidence permits this. Thus, *Depreissia* is the only known cocalodine outside of Australasia. Also, *Depreissia* is the first known non-salticine that strongly resembles ants or wasps.

We note that Deeleman-Reinhold et al. (2016) has also come to the conclusion, independently, that *Depreissia* is related to cocalodines.

## Appendix

**Table:** Locality data for specimens studied morphologically (Figs 2–13).

Species	Locality	Deposited in	Specimen
*Lyssomanes taczanowskii*	Ecuador: Orellana, Yasuní, 0.675°S, 76.398°W	UBC-SEM	ECU11-4328
*Cocalodes papuanus*	New Guinea: Madang, Baiteta forest, 5.02°S 145.75°E	HNHM	
*Depreissia myrmex*	Republic of Congo: Kindamba, Meya, Bangu forest, 3.83°S, 14.5°E	HNHM	Araneae-4515
*Tomocyrba ubicki*	Madagascar: Antsiranana, Parc National de Marojejy, Manantenina River, 14.435°S, 49.76°E	CAS	CASENT9037517
*Hispo sulcata*	Madagascar: Fianarantsoa, Parc Nat. Ranomafana Vohiparara, 21.027°S, 47.45°E	ZMUC	
